# Thrips counts and disease incidence in response to reflective particle films and conservation tillage in cotton and peanut cropping systems

**DOI:** 10.1111/eea.12523

**Published:** 2017-01-03

**Authors:** Ian A. Knight, Glen C. Rains, Albert K. Culbreath, Michael D. Toews

**Affiliations:** ^1^ Department of Entomology University of Georgia 2360 Rainwater Road Tifton GA 31793 USA; ^2^ Department of Plant Pathology University of Georgia 2360 Rainwater Road Tifton GA 31793 USA

**Keywords:** calcium carbonate, kaolin, *Tomato spotted wild virus*, sampling, thrips behavior, Thysanoptera, Thripidae

## Abstract

Feeding damage to seedling cotton and peanut inflicted by adult and immature thrips may result in stunted growth and delayed maturity. Furthermore, adult thrips can transmit *Tomato spotted wilt virus* (TSWV) to seedling peanut, which reduces plant growth and yield. The objective of this research was to assess the efficacy of inert particle films, calcium carbonate or kaolin, in combination with conservation tillage, to reduce adult and immature thrips counts in cotton and peanut crops. Planting cotton or peanut into strip tillage utilizing a rolled rye winter cover crop significantly reduced immature thrips counts. Furthermore, plant damage ratings in cotton as well as TSWV incidence in peanut significantly decreased under conservation tillage. Aboveground cotton biomass and plant stand in cotton and peanut were unaffected by calcium carbonate or kaolin particle film applications. Within each week, immature thrips counts were unaffected by particle films, regardless of application rate. In cotton plots treated with kaolin, total *Frankliniella fusca* (Hinds) (Thysanoptera: Thripidae) counts summed across weeks were significantly greater compared to the untreated control. For adult *F. fusca* counts at 3 weeks after planting, an interaction between tillage and particle film treatments was observed with fewer adult thrips in particle film and strip tillage treated peanut. Similarly, reduced TSWV incidence was observed in particle film‐treated peanut grown using conservation tillage. Neither cotton nor peanut yields were affected by particle film treatments.

## Introduction

Thrips are early‐season pests of cotton and peanut in the southeastern USA. In the spring, thrips disperse from overwintering hosts infesting seedling cotton and peanut (Chamberlin et al., 1992; Northfield et al., 2008; Cook et al., 2011). Symptoms of thrips feeding include stunting, silvering and malformation of the leaves, and damage to or death of the terminal buds (Gaines, 1934; Lynch et al., 1984). Feeding damage to cotton by adult and immature thrips can result in excessive vegetative branching, delayed maturity, and stand loss (Gaines, 1934; Smith & Cothren, 1999; Cook et al., 2011). Peanut is resilient to thrips feeding and damage is largely cosmetic; however, thrips can transmit the economically significant *Tomato spotted wilt virus* (TSWV), which may result in stunting, plant death, and reduced yields (Culbreath et al., 1992, 2003; Groves et al., 2003; Hurt et al., 2005).

Conservation tillage is a practice which loosens soil through a variety of mechanical operations, but endeavors to leave plant residue on the surface (Stinner & House, 1990). By reducing soil disturbance and retaining organic residues, conservation tillage can improve soil organic matter, and reduce erosion, runoff, and leaching of sediments and nutrients (Blevins et al., 1983; Soileau et al., 1994; Liu & Duffy, 1996; Nyakatawa et al., 2001). A variety of arthropod pests is suppressed in conservation tillage systems, while supporting greater densities of natural enemies (Stinner & House, 1990). Increasing ground cover from winter cover crops in conservation tillage systems has been shown to reduce thrips densities and feeding damage in cotton (Bauer & Roof, 2004; Toews et al., 2010). Similarly, thrips densities and TSWV incidence in peanut are reduced under conservation tillage (Johnson et al., 2001; Olson et al., 2006). Conservation tillage with a cover crop, such as rye, reduces soil compaction (Bauer & Busscher, 1996). However, the literature is inconclusive on seedcotton yield benefits when comparing conservation tillage to conventional tillage (Brown et al., 1985; Bauer & Busscher, 1996; Raper et al., 2000). Similarly, peanut performance in conservation vs. conventional tillage is variable and often dependent on effective weed management (Tubbs & Gallaher, 2005).

Various wavelengths in the electromagnetic spectrum are hypothesized to play a significant role in thrips host‐finding behavior. In the visible spectrum, colors such as yellow and white are attractive to thrips and therefore yellow and white sticky traps are often used for monitoring purposes (Walker, 1974; Chu et al., 2000). Teulon et al. (1999) demonstrated that *Frankliniella occidentalis* (Pergande) (Thysanoptera: Thripidae) will fly upwind in the presence of a yellow cue. Ultraviolet light has also been demonstrated to play a significant role in thrips host‐finding behavior. Matteson et al. (1992) found that the ommatidia of *F. occidentalis* had peak sensitivity around 540 nm and in the near ultraviolet (300–400 nm) range. Altering levels of near ultraviolet light has been shown to negatively affect thrips behavior in the laboratory and reduce thrips densities and virus transmission (Costa & Robb, 1999; Costa et al., 2002; Kumar & Poehling, 2006). Conversely, Mazza et al. (1999, 2002) observed that *Caliothrips phaseoli* Hood prefer environments with reduced UVB radiation. The ability to capitalize on thrips sensitivities to ultraviolet light has been demonstrated through the success of UV‐reflective plastic mulches, which reduced thrips densities in field pepper and tomato (Stavisky et al., 2002; Reitz et al., 2003).

Particle films are inert minerals formulated to cover leaf surfaces without interfering with gas exchange or photosynthesis, while reflecting ultraviolet and infrared radiation (Glenn & Puterka, 2005). Particle films have been shown to reduce feeding and oviposition on leaf surfaces by a variety of arthropods (Knight et al., 2000; Lapointe, 2000; Puterka et al., 2000; Liang & Liu, 2002). Feeding, oviposition, and hatch rate of *Thrips tabaci* Lindeman (Thysanoptera: Thripidae) were reduced on onion leaves treated with a kaolin‐based particle film, and reduced thrips densities were observed in kaolin‐treated field plots (Larentzaki et al., 2008). Particle film applications in peanut reduced thrips feeding damage, but did not significantly reduce thrips densities or affect incidence of TSWV. In addition to creating a physical barrier to feeding and oviposition, Glenn et al. (1999) suggested that particle films may visually mask the plants to detection by the thrips. The objective of this study was to evaluate kaolin‐ and calcium carbonate‐based particle films for reducing thrips counts and disease transmission in cotton and peanut grown using conventional and strip tillage systems.

## Materials and methods

Experiments were conducted in 2013 and 2014 at the Coastal Plain Experiment Station, Tift County, GA, USA (cotton: 31°30′54.65″N, 83°32′48.56″W; peanut: 31°31′25.86″N, 83°32′52.89″W) on irrigated fields of Tifton loamy sand (fine‐loamy, kaolinitic, thermic Plinthic Kandiudults). Experiments were independently conducted in cotton and peanut. Treatments included foliar oversprays of particle films over two tillage types. Particle film treatments consisted of an unsprayed control, kaolin (Surround WP; NovaSource, Phoenix, AZ, USA) at low (5.6 kg ha^−1^) and high (11.2 kg ha^−1^) rates, and calcium carbonate (Sombrero; Helena Chemical, Phoenix, AZ, USA) at low (3.1 l ha^−1^) and high (6.3 l ha^−1^) rates. Information about reflected visible light (400–700 nm) was collected from treated leaves of potted cotton plants cultivated in a greenhouse. Tillage components for peanut and cotton consisted of conventional tillage (no residues on soil surface, ground prepared with a disk harrow follow by rip and bed pass) or strip tillage (winter cover crop residues left on soil surface, ground prepared by strip till rig that includes a ripper and tillage in a 25‐cm wide band) into rolled rye. All plots measured 3.7 × 12.2 m and both cotton and peanut rows were planted on 91‐cm row centers.

### Cover crop establishment and seedbed preparation

Prior to planting cover crops in the fall, all plots were disk‐harrowed and then smoothed with a field conditioner. Cover crops for cotton and peanut were planted in late November. Rye (*Secale cereale* L.) cv. Wrens Abruzzi (Poaceae) was planted for all experiments with a Tye Pasture Pleaser no‐till grain drill (AGCO, Duluth, GA, USA) at 101 kg ha^−1^ with a 18‐cm row spacing. Rye cover crops were simultaneously rolled and chemically terminated on 15 April 2013 and 22 April 2014 with 146 ml ha^−1^ flumioxazin (Valor SX; Valent USA, Walnut Creek, CA, USA) tankmixed with 1 606 ml ha^−1^ glyphosate (Roundup WeatherMax; Monsanto, St. Louis, MO, USA). Prior to planting cotton and peanut, tillage plots were tilled with a two‐row strip till rig (Rip/Strip Generation II; Kelly Manufacturing, Tifton, GA, USA) equipped with an in‐row subsoiler adjusted to a depth of 41 cm. Conventional tillage plots were cultivated (Field Cultivator‐Four Bar; Kelly Manufacturing), followed by a rip and bed pass (Rip & Bed‐Generation II; Kelly Manufacturing) with the subsoiler adjusted to a depth of 41 cm.

### Cotton and peanut establishment and management

A four‐row vacuum planter (model 1700 Rigid Integral; John Deere, Moline, IL, USA) equipped with row cleaners was used to sow ‘DP 174RF’ (Deltapine, Memphis, TN, USA) cotton and ‘Georgia‐06G’ peanut on 6 May 2013 and 5 May 2014. All plots were supplemented with overhead irrigation for the entire season. Cotton and peanut plots were managed following recommended standard agronomic practices for Georgia. To alleviate potential confounding effects, late season cotton pests were mitigated with twice‐monthly applications of 292.31 ml ha^−1^ dicrotophos (Bidrin 8; Amvac Chemical, Los Angeles, CA, USA) and 292.31 ml ha^−1^ bifenthrin (Bifen 2 Ag Gold; Direct Ag Source, Eldora, IA, USA) during the bloom cycle. No foliar insecticide applications were made in peanut.

### Particle film applications

Particle films were applied as an aqueous suspension using a CO_2_‐powered backpack sprayer fitted with a two‐row (four‐nozzle) boom sprayer. The boom was equipped with flat fan nozzles (model TP8003E; Spraying Systems, Wheaton, IL, USA) calibrated to deliver 224 l ha^−1^ at 310 kPa. When making applications, the boom was centered between the two rows at a distance of 30 cm above the plant canopy. Particle films required manual agitation in the field to prevent settling and clogging of the nozzles. The first particle film applications to cotton were made on 15 May 2013 and 13 May 2014, at full emergence. Reapplications were made to cotton on 19, 23, and 28 May and 3 June 2013, and on 16, 23, and 30 May 2014 to account for plant growth, irrigation, and rainfall. The first particle film applications to peanut were made on 19 May 2013 and 16 May 2014, at full emergence. Reapplications were made to peanut on 23 and 28 May and 3, 7, and 9 June 2013, and on 23 and 30 May 2014.

### Collection of film reflectance

Potted cotton plants were grown in a greenhouse. At full emergence the pots were placed outside on the ground and treated with a single application of particle films following the same methods as field applications. Once films dried, reflectance data between 400 and 700 nm were collected with an OceanOptics S2000 Spectrometer provisioned with an LS‐1 tungsten‐halogen lamp light source and R200‐VIS‐IR Standard Reflection probe (OceanOptics, Dunedin, FL, USA) positioned 3 mm from the leaf surface in a clamp to exclude confounding light. Three plants per treatment were scanned with six scans per plant for a total of 18 scans per treatment to characterize the spectral profile for each particle film treatment. Reflectance data were collected as percent reflectance of light relative to a white Teflon standard. The actual reported value for each individual scan was the mean of 30 scans in the same exact leaf area, each lasting 150 ms. The scanner software was programmed to utilize a boxcar width of 5.

### Thrips sampling

Thrips were sampled from cotton plots at 7, 14, and 21 days after emergence from five randomly selected plants in each plot. Cotton plants were removed from the soil and inverted into a 0.47‐l glass jar filled with 100 ml of 70% ethyl alcohol. While immersed in the alcohol, plants were vigorously shaken to dislodge the thrips and then the plants were discarded. Resulting thrips specimens were transported to the laboratory for enumeration and identification. In the laboratory, contents from each jar were carefully rinsed through a fine‐mesh sieve (125‐μm openings) and transferred to 20‐ml vials with 70% ethyl alcohol. Peanuts were sampled for thrips at 10, 17, and 24 days after emergence. The terminal bud from each of five plants per plot was removed and placed in a 20‐ml vial filled with 10 ml of 70% ethyl alcohol. In the laboratory, thrips were separated by life stage, identified to species (adults only), and enumerated under a dissecting microscope (60× magnification).

### Plant height and leaf count

Plant height and true leaf count were assessed in cotton plots at 7, 14, and 21 days after emergence. Five plants were randomly selected from each plot and measured from the ground to the terminal to assess plant height. Leaf counts were determined from the number of true leaves (>2 cm diameter) on five plants randomly selected from each plot.

### Plant stand, plant biomass, and *Tomato spotted wilt virus* ratings

Plant stand was determined from a representative row from each plot for cotton and peanut. In cotton, plant stand was determined by enumerating main stems in a single representative row at 4 weeks after emergence. In peanut, plant stand was determined by enumerating all tap roots in a representative row after digging the peanuts in preparation for harvest. Additional response variables were taken for cotton only. Dry plant biomass was evaluated by clipping five random plants per plot at the soil surface and drying them in a laboratory oven for 48 h at 50 °C prior to weighing on a laboratory balance. At 1 week prior to harvest (134 days after planting), TWSV incidence was assessed. Incidence was determined by the percent of 0.3‐m portions of row containing one or more symptomatic plants. Symptoms that were included as a positive TSWV hit included any of the following: severe stunting, characteristic ring spots, ‘oak leaf’ chlorosis patterns, wilting, or death (Culbreath et al., 1997).

### Yield determination

Peanuts were dug and inverted on 24 September 2013 and 25 September 2014 and the center two rows from each four‐row plot were harvested using a pull‐type peanut combine on 1 October 2013 and 2 October 2014. Individual bags of whole pods were weighed and adjusted to a 10%‐moisture standard. After weighing, 500‐g samples from each yield sample were submitted for grading, performed by the Tifton Lab Facility of the Georgia Department of Agriculture, a USDA‐GIPSA‐FGIS licensed facility. Total Sound Mature Kernels (TSMK) and Other Kernels (OK) were the grade factors used for comparison. Seedcotton was harvested on 2 October 2013 and 2 October 2014 from the center two rows of individual cotton plots with a two‐row spindle picker. Lint yield was estimated from seedcotton weights at 39% gin turnout.

### Statistical analysis

Trials were arranged in randomized complete blocks with a two‐way factorial treatment structure with four replicates per treatment combination. Cotton and peanut were analyzed independently. Data were modeled by week of collection (when appropriate) using Proc GLIMMIX (SAS Institute, 2011) with block designated as the random variable. All response variables, except for thrips counts, were modeled with a Gaussian distribution. Thrips counts were modeled with a negative binomial distribution because the observed sample variance of the discrete data exceeded the sample mean in an unbounded positive range. For detecting differences among treatments (α = 0.05), treatment means were separated following the LSMEANS procedure. All reported means were back‐transformed using the ILINK function and back‐transformed means and standard errors are presented in the text and on all figures. Spectrometer reflectance data were jackknifed to every 10 nm between 400 and 700 nm to facilitate analysis. Statistical comparisons among particle film treatments were conducted on the percent reflectance response variable. Those statistics were derived from an ANOVA (Proc GLIMMIX) with a Tukey's honestly significant difference (HSD) adjustment for separating treatment means. Reflectance standards for each particle film treatment were correlated with mean total adult *F. fusca* using Proc CORR (SAS Institute, 2011).

## Results

### Cotton

#### Thrips sampling

A total of 1 984 adult thrips and 4 190 immature thrips were enumerated from cotton plots in 2013 and 1 771 adult thrips and 7 172 immature thrips in 2014. Adult thrips species composition in 2013 consisted of 90.0% *F. fusca*, 5.4% *F. occidentalis*, 2.7% *Frankliniella tritici* (Fitch), 1.7% *Frankliniella bispinosa* (Morgan), and <1.0% *T. tabaci*. Adult thrips in 2014 were 77.6% *F. fusca*, 22.0% *F. occidentalis*, 0.2% *F. tritici*, and 0.2% *T. tabaci*.

**Figure 1:**
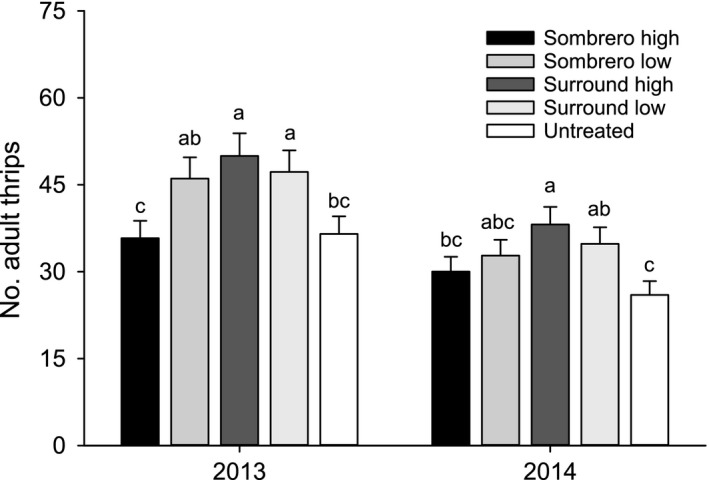
Mean (+ SE) sum of adult *Frankliniella fusca* across weeks in 2013 and 2014 in cotton treated with particle film. Means within a year capped with the same letter are not significantly different (LSMEANS test: P>0.05).

There was no interaction between tillage and particle film treatment on adult thrips counts on any sample week in 2013 or 2014. However, significantly fewer adult thrips were observed in strip tillage plots on each sample week in 2013 (F_1,30_ = 6.46–39.61, P≤0.001–0.016) and 2014 (F_1,30_ = 8.56–52.48, P≤0.001–0.006) (Table 1). Total adult thrips counts were unaffected by particle film treatments on any sample date. However, when only *F. fusca* adult thrips counts were summed across weeks, the effect of particle film treatment on the total number of adult *F. fusca* collected from plots over the course of this study was significant in both years (2013: F_4,30_ = 3.61, P = 0.016; 2014: F_4,30_ = 2.89, P = 0.039; Figure 1).

**Table 1:** Mean (± SE) number of adult thrips by tillage type and sample date

Year	Sample date	Tillage type	
		Conventional	Rolled rye
2013	22 May	22.4 ± 2.0b	13.9 ± 0.9a
	28 May	29.2 ± 2.2b	14.1 ± 1.2a
	5 June	10.4 ± 1.1b	7.1 ± 0.9a
2014	20 May	23.6 ± 1.9b	9.5 ± 0.9a
	27 May	10.3 ± 1.3b	5.2 ± 0.7a
	3 June	23.0 ± 2.1b	15.5 ± 1.5a

Means within a row followed by different letters are significantly different (LSMEANS test: P<0.05).

**Figure 2:**
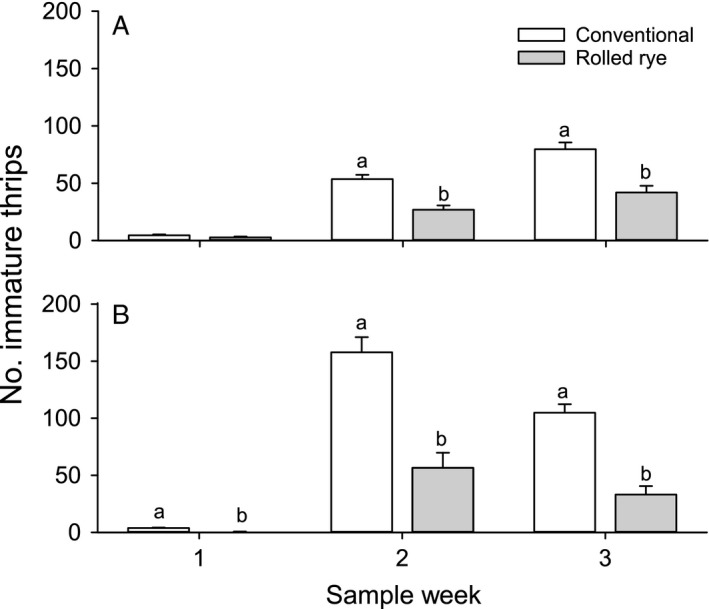
Mean (+ SE) total number of immature thrips per five whole cotton seedlings by tillage type for each sample week of (A) 2013 and (B) 2014. Means within a sample week and year capped with different letters are significantly different (LSMEANS test: P<0.05).

Immature thrips counts varied based on week of study. There was no interaction between tillage and particle film treatment on any sample date of either year. There were also no effects of particle film treatment on immature thrips counts. Immature thrips counts did not significantly differ by tillage type on the first sample date of 2013, but they were reduced in the strip tillage plots on the second (F_1,30_ = 41.08, P<0.001) and third sample dates of 2013 (F_1,30_ = 30.34, P<0.001) and each sample date in 2014 (F_4,30_ = 28.94–47.11, P<0.001) (Figure 2).

#### Particle film reflectance and adult *Frankliniella fusca* counts

**Figure 3:**
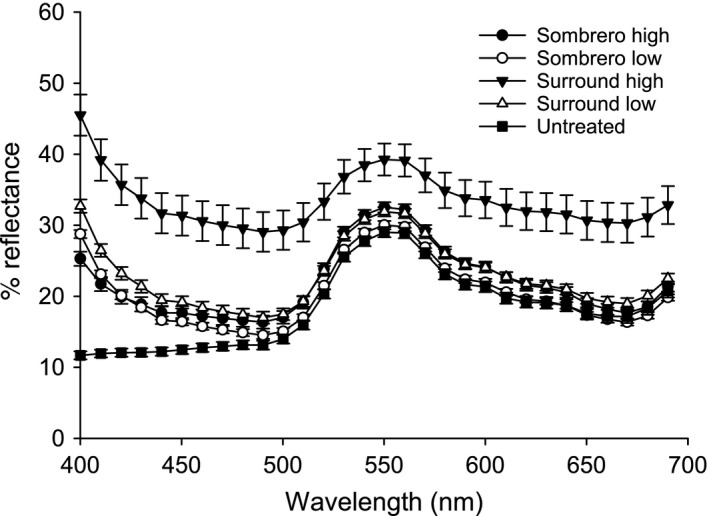
Mean (± SE) reflectance (%) at selected wavelengths of particle film‐treated cotton seedling leaves.

Data for the reflection of light between 400 and 700 nm are expressed as percentage reflectance of a polished white Teflon standard (Figure 3). Reflectance of the high rate of Surround WP was significantly greater than all other treatments at each selected wavelength (F_4,78_ = 11.31–66.84, P<0.001). At 400 nm, the low rate of Surround WP was significantly greater than the high rate of Sombrero and untreated leaves, whereas both rates of Sombrero were greater than the untreated; however, none of the latter were significantly different from each other (F_4,78_ = 66.84, P<0.001). At 500 nm, only the high rate of Surround WP differed from the remaining treatments (F_4,78_ = 18.78, P<0.001). When mean adult *F. fusca* counts summed across weeks for each particle film treatment were correlated with reflectance standards at each wavelength, counts were positively correlated with % reflectance at 400 nm in both years. The correlation coefficients were marginally non‐significant in 2013 (r = 0.84, P = 0.073; n = 5), and significant in 2014 (r = 0.98, P = 0.002; n = 5).

#### Estimates of plant height and true leaf counts

The extent that tillage and particle film treatments affected plant height varied by week. Within weeks, there were no tillage*particle film interactions in either year. Plant heights on the first two sample dates of 2013 (F_1,30_ = 31.01–71.29, P<0.001) and 2014 (F_1,30_ = 5.5–15.11, P≤0.001–0.026) were significantly affected by tillage type, but did not significantly differ by tillage by the third week (Table 2). Particle film treatments had no effect on plant height on any sample date in either year.

**Table 2:** Mean (± SE) plant height (cm) by tillage type and sample date

Year	Sample date	Tillage type	
		Conventional	Rolled rye
2013	22 May	5.10 ± 0.12b	6.50 ± 0.12a
	28 May	8.29 ± 0.17b	9.72 ± 0.19a
	5 June	14.62 ± 0.23a	15.20 ± 0.37a
2014	20 May	4.69 ± 0.10b	5.44 ± 0.15a
	27 May	7.87 ± 0.21b	8.43 ± 0.25a
	3 June	12.78 ± 0.37a	13.56 ± 0.38a

Means within a row followed by different letters are significantly different (LSMEANS test: P<0.05).

Treatment effects on leaf count were not consistent, and true leaves that met the sampling criteria were not present by the first sample date of 2014. There was no tillage*particle film interaction on true leaf counts on any sample date of either year. Conservation tillage significantly reduced true leaf counts compared to conventional tillage on the first and third sample date [22 May 2013, conventional: mean ± SE = 0.8 ± 0.1, rolled rye: 0.5 ± 0.1, F_1,27_ = 11.24, P = 0.002; 5 June 2013, conventional: 4.6 ± 0.1, rolled rye: 3.9 ± 0.1, F_1,27_ = 67.16, P<0.001], but not on the second sample date (28 May 2013, conventional: 2.1 ± 0.1, rolled rye: 2.0 ± 0.0; F_4,30_ = 2.57, P = 0.12). There were no true leaves on the first sample date of 2014. Tillage effects were not significant for leaf count in any week of 2014. Regardless of sample date or year, there was no effect of particle film treatment on true leaf counts.

#### Plant stand, dry biomass, and harvest weight estimates

Four weeks after full emergence, there were no significant interactions between tillage and particle film treatment in the cotton stand in either year (2013: F_4,30_ = 2.69, P = 0.052; 2014: F_4,30_ = 1.67, P = 0.18). Tillage effects were significant in both years, with more plants in conventional than in strip tillage (2013, conventional: mean ± SE = 89.7 ± 2.7, rolled rye: 73.6 ± 3.1, F_1,30_ = 16.74, P<0.001; 2014, conventional: 95.8 ± 2.1, rolled rye: 88.7 ± 3.0, F_1,30_ = 4.61, P = 0.040). Particle film treatment alone did not affect plant stand (2013: F_4,30_ = 0.93, P = 0.46; 2014: F_4,30_ = 2.66, P = 0.052).

No significant interaction between tillage and particle film treatment on dry weight biomass was noted in either year. Tillage only had an effect on biomass in 2013, with conventional tillage exhibiting greater dry biomass than strip tillage (13.88 ± 0.57 vs. 10.44 ± 0.49 g; F_1,30_ = 20.63, P<0.001). Mean dry weight in 2014 was 39.16 ± 1.97 g, and was unaffected by tillage. Particle film treatment had no significant effect on dry biomass in either year.

Harvest weight was unaffected by a tillage*particle film interaction in either year, nor was there a significant effect of particle film treatment in either. Harvest weight was lower in conservation tillage plots for both years of the study (2013, conventional: 1 361.6 ± ± 56.7 kg ha^−1^, rolled rye: 1 044.0 ± 47.9 kg ha^−1^, F_1,30_ = 14.93, P<0.001; 2014, conventional: 1 270.6 ± 40.9 kg ha^−1^, rolled rye: 1 108.0 ± 29.6 kg ha^−1^, F_1,30_ = 11.54, P = 0.002).

### Peanut

#### Thrips sampling

In total 178 adult thrips and 8 282 immature thrips were enumerated from peanut leaflets in 2013 and 725 adult thrips and 11 343 immature thrips in 2014. Species composition of adult thrips was not assessed in 2013, but in 2014 adult thrips species composition was 96.5% *F. fusca*, 1.7% *F. occidentalis*, and 1.7% *F. tritici*.

**Figure 4:**
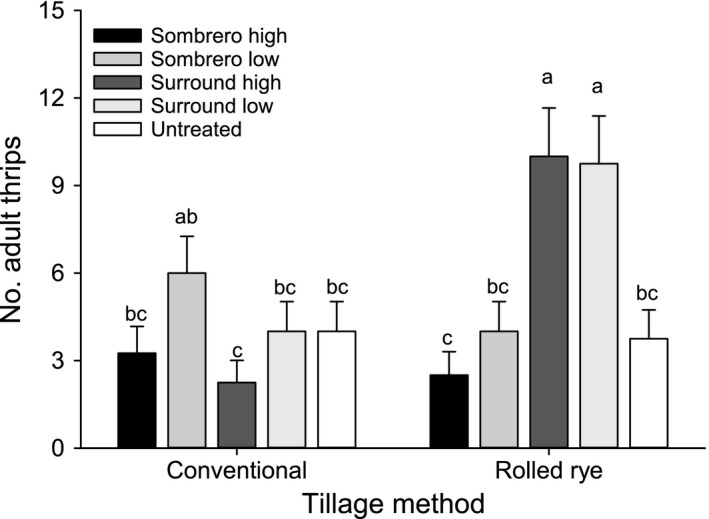
Mean (+ SE) sum of adult thrips in peanut treated with tillage and particle film for the 2nd week of sampling in 2014. Means capped with the same letter are not significantly different (LSMEANS test: P>0.05).

Tillage and particle film treatments caused few significant effects on adult thrips counts. No tillage*particle film interactions were observed on any sample date in 2013. No significant interactions were observed on the first and last sample dates of 2014; however, a tillage*particle film interaction was significant for adult thrips on the second sample date of 2014 (F_4,30_ = 5.23, P = 0.003) (Figure 4). Tillage was not significant during any week of sampling in either year (2013: F_1,30_ = 0–1.56, P = 0.56–0.96; 2014: F_1,30_ = 0.34–4.13, P = 0.051–0.56). No significant effect attributed to particle film treatment was observed in either year (29 May 2013, conventional: mean ± SE = 11.38 ± 1.49, rolled rye: 8.25 ± 1.49; 5 June 2013, conventional: 0.68 ± 0.22, rolled rye: 1.11 ± 0.24; 12 May 2013, conventional: 0.06 ± 4.67, rolled rye: 2.23 ± 0.41).

**Figure 5:**
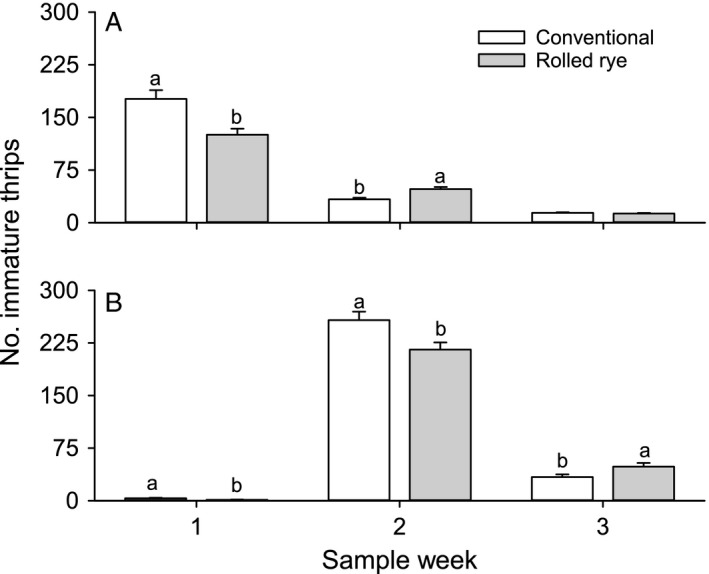
Mean (+ SE) total number of immature thrips per five peanut leaf buds by tillage type for each sample week of (A) 2013 and (B) 2014. Means within a sample week and year capped with different letters are significantly different (LSMEANS test: P<0.05).

No tillage*particle film interactions on immature thrips counts were observed in either year (2013: F_4,30_ = 0.45–2.27, P = 0.084–0.77; 2014: F_4,30_ = 0.48–1.74, P = 0.17–0.75). The effect of tillage on immature thrips counts was significant on the first two (F_1,30_ = 11.94–14.75, P = 0.001–0.002), but not on the third sample date in 2013. Tillage effects were significant for immature thrips on each week of sampling in 2014 (F_1,30_ = 5.26–8.57, P = 0.006–0.029; Figure 5). Particle films had no effect on immature thrips counts in 2013, or at the 1st and 3rd weeks of sampling in 2014. On the second sample date in 2014, plots treated with high rates of Surround WP had significantly more immature thrips than the low rate of Sombrero or untreated control, though no other combinations of treatments differed (Sombrero High: 245.7 ± 18.2, Sombrero Low: 224.7 ± 16.7, Surround High: 281.7 ± 20.7, Surround Low: 231.2 ± 17.2, untreated: 202.2 ± 15.1; F_4,30_ = 2.72, P = 0.048).

#### *Tomato spotted wilt virus*, plant stand, harvest weight, and grading

**Figure 6:**
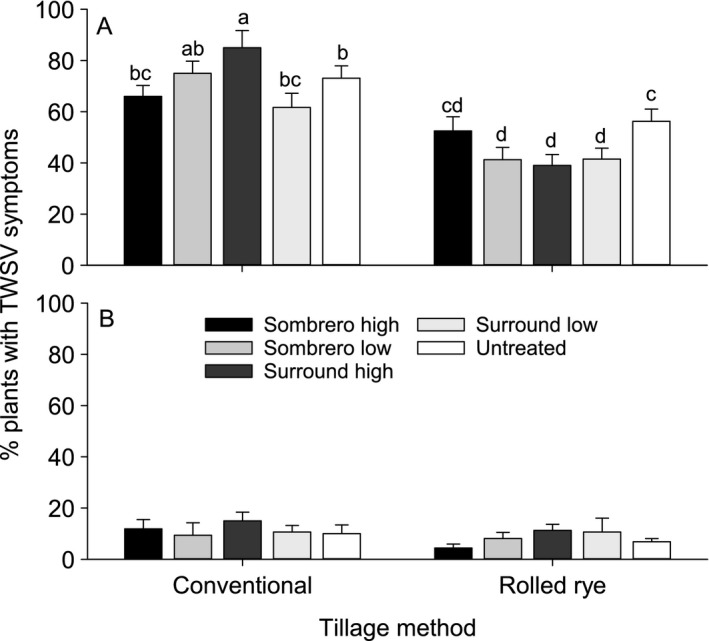
Mean (+ SE) percentage of *Tomato spotted wilt virus*‐infected 0.3‐m segments per row treated with tillage and particle film at 1 week prior to harvest in (A) 2013 and (B) 2014. Means capped with different letters are significantly different (LSMEANS test: P<0.05).

A significant interaction between tillage and particle film treatment for TSWV incidence was recorded prior to harvest in 2013 (F_4,30_ = 4.85, P = 0.004; Figure 6). Tillage main effects were only significant for TSWV incidence in 2013 (2013: F_1,30_ = 75.59, P<0.001; 2014: F_1,30_ = 2.21, P = 0.15). There was no effect of particle film treatment on TSWV incidence in either year (2013: F_4,30_ = 2.38, P = 0.074; 2014: F_4,30_ = 0.79, P = 0.54).

Plant stand as measured by taproots unearthed after digging was unaffected by a tillage by particle film interaction in either year (2013: F_4,30_ = 0.45, P = 0.77; 2014: F_4,30_ = 0.37, P = 0.57). Plant stand was significantly lower in conventional tillage compared to rolled rye in 2013 (conventional: mean ± SE = 60.4 ± 2.5; rolled rye: 69.0 ± 2.0; F_1,30_ = 6.71, P = 0.015), but not in 2014 (conventional: 98.2 ± 2.5; rolled rye: 103.2 ± 2.6; F_1,30_ = 1.9, P = 0.18). No significant differences in plant stand by particle film treatment were observed in either year (2013: F_4,30_ = 0.66, P = 0.62; 2014: F_4,30_ = 0.95, P = 0.45).

**Figure 7:**
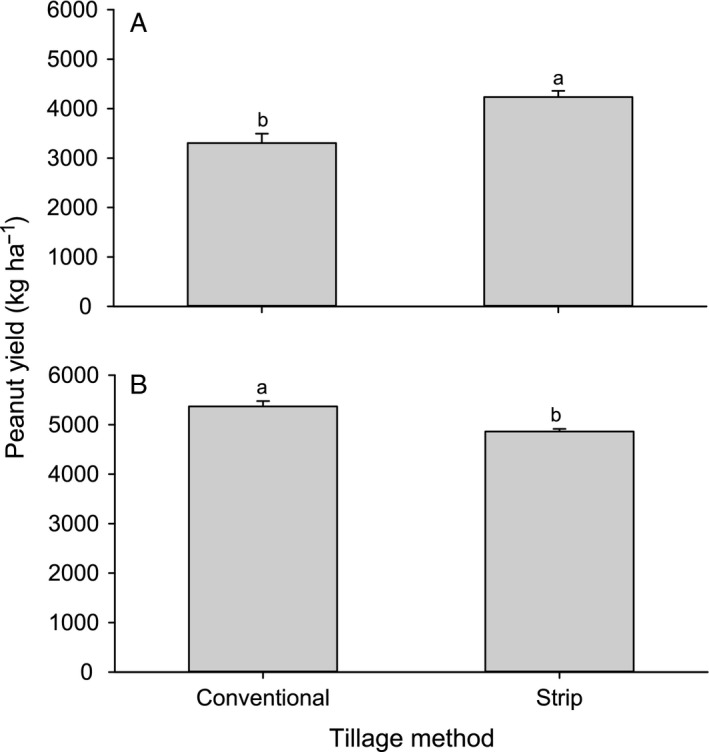
Mean (+ SE) in‐pod peanut yield (kg ha^−1^) by tillage type in (A) 2013 and (B) 2014. Means within years with different letters are significantly different (LSMEANS test: P<0.05).

No effect of tillage*particle film interaction on yield as measured by in‐shell harvest weights was observed in either year (2013: F_4,30_ = 1.12, P = 0.37; 2014: F_4,30_ = 0.6, P = 0.67). Yield was greater in strip compared to conventional tillage in 2013 (F_4,30_ = 18.38, P<0.001), whereas the reverse was true for 2014 (F_4,30_ = 16.93, P<0.001; Figure 7). Particle film treatments had no effect on yield in either year (2013: F_4,30_ = 1.5, P = 0.23; 2014: F_4,30_ = 1.14, P = 0.36). Mean total sound mature kernel proportion was 0.700 ± 0.003, and there were no significant interaction (F_4,30_ = 0.14, P = 0.97) or main effects of tillage (F_4,27_ = 0.79, P = 0.38) or particle film treatments (F_4,30_ = 0.39, P = 0.81).

## Discussion

Particle films are not supposed to interfere with photosynthesis or gas exchange (Glenn & Puterka, 2005). Weekly plant height and leaf count data, as well as plant stand, biomass, and yield data from cotton all indicate that neither kaolin nor calcium carbonate‐based particle films impacted plant growth, despite repeated applications to seedlings. Similarly, the lack of differences in taproot counts, yield, and grade demonstrates that peanut growth was unaffected by particle film treatments.

Beneficial effects of particle films suppressing thrips were not observed for either cotton or peanut. For cotton, there were no weekly effects of particle film for either adults or immature counts. In peanut, there were two instances in 2014 where significantly more adult or immature thrips were observed in kaolin‐treated compared with the untreated plots. Interestingly, a similar trend emerges when the total number of *F. fusca* were summed across weeks whereby significantly more thrips were enumerated from high‐ and low‐rate kaolin plots compared to untreated controls. Larentzaki et al. (2008) observed reduced thrips counts, feeding, and reproductive success of *T. tabaci* on kaolin‐treated onions and similar results have been observed with respect to feeding and reproduction for a variety of insect pests (Lapointe, 2000; Liang & Liu, 2002; Showler, 2002; Saour, 2005). Here, neither kaolin nor calcium carbonate particle films prevented colonization by adult thrips, or deterred thrips reproduction on the seedling cotton or peanut.

The reflective properties of the particle film treatments varied by wavelength and application rate. Only the high rate of Surround WP was consistently different from the other treatments. Reflectance of all particle films was greatest around 400 nm. The observed correlation between total adult *F. fusca* and % reflectance at 400 nm suggests that wavelengths in this range and lower (wavelengths in the ultraviolet range) should be further investigated for effects on thrips host finding or selection. Both kaolin and calcium carbonate gave the plants a white hue to the naked eye and previous studies demonstrated that white is attractive to *F. fusca* (Walker, 1974; Chu et al., 2000), whereas violet traps are attractive to *F. occidentalis* (Vernon & Gillespie, 1990). In 2014, weekly reflectance data were collected from the field using a Fieldspec3 spectroradiometer (ASD, Boulder, CO, USA); although there were no significant differences in reflectance between particle films, strip tilled plots were significantly less reflective than conventional tillage (Ian Knight, unpubl.).

The spectrometer used in this study would not allow for accurate analysis of reflectance below 400 nm; however, Glenn et al. (2002) observed that Surround WP applied to apples significantly increased reflection of UV‐A (320–400 nm). In that study the highest rate of Surround consistently reflected more UV‐B than the untreated control. There is no peer reviewed literature regarding the ultraviolet reflectance of Sombrero or other calcium carbonate particle films. Walker (1974) and Mazza et al. (1999, 2002) suggest that surfaces and environments with reduced ultraviolet reflection were more attractive to thrips. Rates of particle films used in this study may have been insufficient to reflect sufficient UV light to deter thrips colonization.

The interaction between tillage and particle film treatments on pre‐harvest TSWV incidence in 2013 peanut plots suggests that the particle films may play a role in reducing thrips feeding. A potential explanation is that minor reductions in thrips feeding as a result of particle film treatments were apparent under the reduced thrips counts in conservation tillage, whereas these effects were overwhelmed by large thrips counts on conventionally tilled peanut. Wilson et al. (2004) also showed that thrips counts were unaffected by application of particle film, but thrips feeding damage was reduced. An alternative explanation, which accounts for the interactions between tillage and particle film treatment with respect to feeding damage and disease transmission, is that the particle films stimulated additional movement among thrips but the suppressive effects of the strip tillage mitigated the additional feeding.

The overall value of strip tillage was variable between cotton and peanut systems. Cotton stand establishment was an issue in strip tillage cotton; plant stands were lower despite significantly reduced thrips counts and feeding damage. Despite the difficulty of establishing cotton in heavy rye residue, stand counts were all within the 3–15 plants m^−2^ range where differences in yield are not expected (Bednarz et al., 2000; Boquet et al., 2004). Moreover, multiple studies have demonstrated comparable or improved cotton yield under conservation tillage (Brown et al., 1985; Schwab et al., 2002; Boquet et al., 2004; Balkcom et al., 2007). Increased nitrogen immobilization and volatilization under conservation tillage can be overcome with a supplemental fertilizer application (Gilliam & Hoyt, 1987). Although Georgia Cooperative Extension recommends additional nitrogen at side dress in newly converted strip tilled fields (Collins et al., 2014), no additional nitrogen was applied to allow for direct comparisons with the conventional tilled plots. Ironically, differences in nitrogen availability to the cotton may have contributed to observed reductions in biomass and yield as the decaying rye residue could have bound nutrients including nitrogen.

The difference in the effects of tillage on peanut yield between years was likely due to the differences in TSWV incidence. The results of this study resemble those of Marois & Wright's (2003) who reported a decrease in TSWV severity and a subsequent increase in yield in peanut plots strip tilled into wheat cover, but no significant differences in TSWV or yield the following year. Appropriate weed management has been shown to be an important consideration for peanuts grown under conservation tillage (Tubbs & Gallaher, 2005; Price et al., 2007). Weeds were not managed differently between tillage types for either cotton or peanut, and no issues with weeds were noted over the course of this study.

The reduction in thrips counts under conservation tillage on seedling cotton and peanut reported here are consistent with reports from similar studies (Olson et al., 2006; Toews et al., 2010). Due to the very high number of thrips infesting seedling peanut, sampling leaflets is a more efficient means of collecting data on thrips counts compared to whole plants. However, this method did not provide an adequate sampling of adult thrips. As a result, adult counts might be inferred from immature thrips counts and disease transmission. Because TSWV is transmitted by adult thrips, the reduction in immature thrips and the reduced disease incidence observed under strip tillage strongly suggest that despite non‐significant results, adult thrips counts may have been lower in the strip tillage plots (Groves et al., 2001).

Although the authors intended to use the particle films to alter the reflectance and thereby mask cotton plants from detection by the thrips, both kaolin and calcium carbonate are labeled for use on cotton. Surround WP is marketed for thrips suppression at 28–56 kg of material diluted in 935 l of water ha^−1^; conversely, instructions for application through a backpack sprayer suggest mixing at 30–60 g of material per l of water. Sombrero is labeled as a solar protectant on cotton at 9.4–18.7 l of material diluted in 62–372 l of water ha^−1^. Here, there were no observed reductions in thrips population or yield preservation in cotton or peanut attributed to particle film usage. Applying these materials required constant agitation to prevent settling, considerably more water than conventional pesticides for application, repeated applications in the event of rainfall and plant growth, and frequent cleaning of clogged filters and nozzles. Combined with the lack of efficacy, these attributes strongly suggest that particle films are unlikely to gain usage for thrips management in row crop systems like cotton or peanut.
